# Stereoselective Synthesis and Cytoselective Toxicity of Monoterpene-Fused 2-Imino-1,3-thiazines

**DOI:** 10.3390/molecules191015918

**Published:** 2014-10-02

**Authors:** Zsolt Szakonyi, István Zupkó, Reijo Sillanpää, Ferenc Fülöp

**Affiliations:** 1 Institute of Pharmaceutical Chemistry, University of Szeged, Eötvös utca 6, H-6720 Szeged, Hungary; 2 Department of Pharmacodynamics and Biopharmacy, University of Szeged, Eötvös u. 6, H-6720 Szeged, Hungary; E-Mail: zupko@pharm.u-szeged.hu; 3 Department of Chemistry, University of Jyväskylä, P.O. Box 35, 40351 Jyväskylä, Finland; E-Mail: e.reijo.j.sillanpaa@jyu.fi; 4 Stereochemistry Research Group of the Hungarian Academy of Sciences, Eötvös u. 6, H-6720 Szeged, Hungary

**Keywords:** monoterpene, 1,3-amino alcohol, stereoselective, CDI, 1,3-thiazine, antiproliferative

## Abstract

Starting from pinane-, apopinane- and carane-based 1,3-amino alcohols obtained from monoterpene-based β-amino acids, a library of monoterpene-fused 2-imino-1,3-thiazines as main products and 2-thioxo-1,3-oxazines as side-products were prepared via two- or three-step syntheses. When thiourea adducts prepared from 1,3-amino alcohols and aryl isothiocyanates were reacted with CDI under mild conditions, O-imidazolylcarbonyl intermediates were isolated which could be transformed to the desired 1,3-thiazines under microwave conditions. 1,3-Thiazines and 2-thioxo-1,3-oxazine side-products could also be prepared in one-step reactions through the application of CDI and microwave irradiation. The ring-closure process was extended to cycloalkane-based γ-hydroxythioureas. The carane- and apopinane-based derivatives exhibited marked antiproliferative activity against a panel of human adherent cancer cell lines (HeLa, A2780, MCF7 and A431).

## 1. Introduction

In the past decade, alicyclic 1,3-amino alcohols have proved to be versatile building blocks. They have been applied as useful starting materials in the stereoselective syntheses of compounds of pharmacological interest and they have served as chiral ligands and auxiliaries in enantioselective transformations [1,2,3].

Several natural chiral terpenes, including (+)-pulegone [4,5,6] α- and β-pinene [7,8,9] and fenchone-camphor [10,11,12], have proved to be excellent sources for the production of various amino alcohols, which have been successfully applied in enantioselective syntheses. The transformation of enantiomerically pure α-pinene to β-amino acid derivatives such as 1,3-amino alcohols was recently reported [9,13]. These synthons served as chiral auxiliaries in the enantioselective synthesis of secondary alcohols or pharmacons, e.g., esomeprasol [14,15,16,17,18].

Besides their value in enantioselective catalysis, 1,3-amino alcohols are good starting points for the synthesis of various heterocyclic ring systems, such as 1,3-oxazines, 1,3-thiazines or 1,4-oxazepams [2,19]. The 2-imino-1,3-thiazine and 2-iminothiazolidine ring systems can be found as moieties in biologically relevant compounds, including antifungicidal and antimicrobial agents [20], BACE1 inhibitors [21], or cannabinoid receptor agonists [22,23,24]. New spiro derivatives of 2-imino-1,3-thiazines have been synthetized and shown to be potential neuroprotectors [25].

In recent years, we have devised novel pathways to synthetize new monoterpene-based chiral β-lactams and β-amino acid derivatives derived from (–)- and (+)-α-pinene, (–)-3-carene, (–)- and (+)-apopinene and myrtenic acid [9,13,26,27,28,29,30]. These amino acid derivatives have proved to be excellent building blocks for the syntheses of compounds with multidrug resistance (MDR) antagonist activity [29].

We also found that monoterpene-based 1,3-amino alcohols prepared from the abovementioned β-amino acid derivatives are excellent building blocks for the synthesis of 2-imino-1,3-oxazines which possess marked anti-cancer activity [31]. The analogous pinane- or apopinane-based 2-imino-1,3-thiazines could not be prepared by the methods applied earlier for the synthesis of cycloalkane- or norbornane-fused analogue 1,3-thiazines [32,33].

The aim of the present work was to synthetize new chiral pinane-, apopinane- and carane-fused 2-imino-1,3-thiazines, analogues of 1,3-oxazines with noteworthy cytoselective toxicity on multiple cancer cell lines.

## 2. Results and Discussion

### 2.1. Syntheses of Alicyclic and Monoterpene-Based 1,3-Amino Alcohols

**Figure 1:**
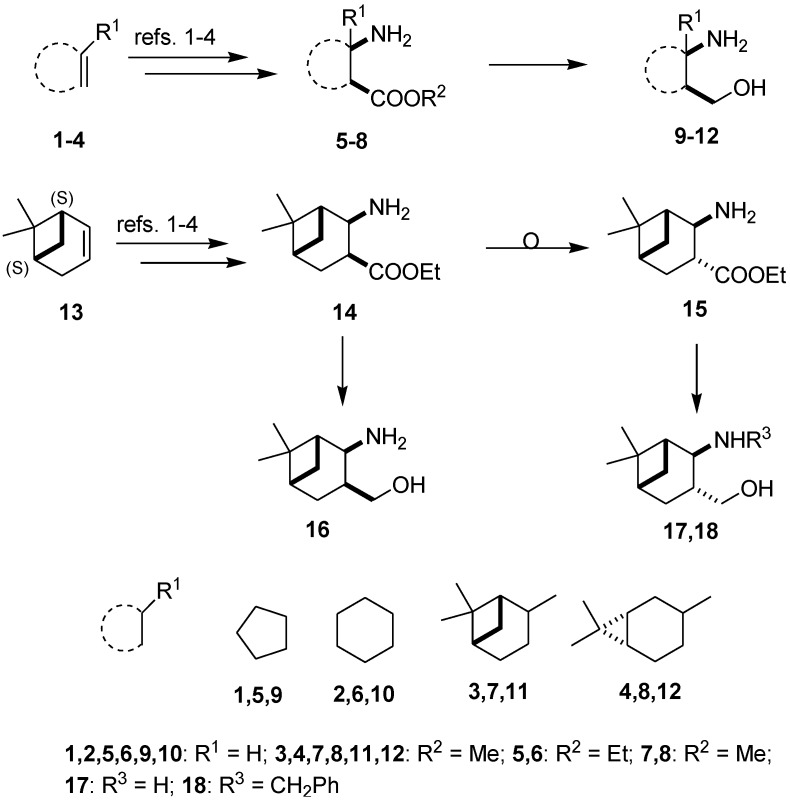
Synthesis of 1,3-amino alcohol starting materials.

The synthetic routes applied for the preparation of 1,3-amino alcohols **9**–**12** and **16**–**18** followed literature methods (Figure 1) [9,13,27,28,29,31,33]. The corresponding β-lactams were prepared by the stereoselective cycloaddition of chlorosulfonyl isocyanate to cyclopentene, cycloxene, α-pinene, 3-carene and apopinene, followed by ring opening, which resulted in *cis*-fused β-amino esters **5**–**8** and **14**. Under alkaline conditions, the *cis*-amino ester **14** underwent fast and complete isomerization at the carboxylic function, resulting in the *trans*-amino ester **15** in excellent yield [28]. Reduction of **5**–**8**, **14** and **15** with LAH led to the primary amino alcohols **9**–**12**, **16** and **17**. From **17**, *N*-benzyl derivative **18** was prepared by reductive alkylation with benzaldehyde and NaBH_4_ in EtOH [31].

### 2.2. Syntheses of 2-Imino-1,3-thiazine Derivatives

**Scheme 1:**
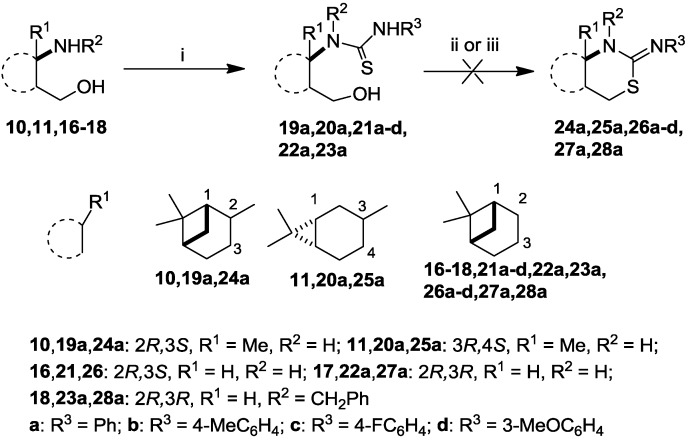
Synthesis and attempted ring closure of thioureas **19**–**23**.

The intermediate thiourea adducts **19**–**23** were prepared in good to excellent yields by the reaction of the appropriate aryl isothiocyanates and 1,3-amino alcohols **10**, **11** and **16**–**18** (Scheme 1, Table 1) [13,27,31].

*Reagents and conditions*: (i) 1.05 equiv. R^3^NCS, toluene, rt, yield: 85%–95%; (ii) 10% HCl/EtOH, rt to reflux; (iii) DEAD, TPP, THF, rt.

**Table 1:** Thioureas **19**–**23** and *O*-imidazolylcarbonyl intermediates **29**–**33**.

General Structure	R^1^	R^2^	R^3^	Compound No.
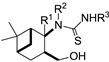	Me	H	Ph	**19a**
	H	H	Ph	**21a**
	H	H	4-MeC_6_H_4_	**21b**
	H	H	4-FC_6_H_4_	**21c**
	H	H	3-MeOC_6_H_4_	**21d**
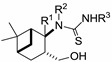	H	H	Ph	**22a**
	H	CH_2_Ph	Ph	**23a**
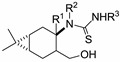	Me	H	Ph	**20a**
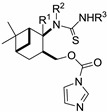	Me	H	Ph	**29a**
	H	H	Ph	**31a**
	H	H	4-MeC_6_H_4_	**31b**
	H	H	4-FC_6_H_4_	**31c**
	H	H	3-MeOC_6_H_4_	**31d**
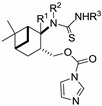	H	H	Ph	**32a**
	H	CH_2_Ph	Ph	**33a**
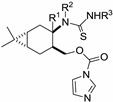	H	H	H	**30a**

Although a number of methods are known for the conversion of 3-hydroxypropylthioureas to the corresponding 2-imino-1,3-thiazines, e.g., acid-promoted dehydrative cyclization [32,33] or Mitsubobu conditions [34], in the cases of the highly constrained acid-sensitive bicyclic pinane and carane skeletons the expected 2-imino-1,3-thiazine systems were not obtained.

Bernacki *et al.* recently reported that they also identified 2-aminothiazoline cyclization products besides the expected main product thioureas in the reactions of 1,2-amino alcohols and 3-aminobenzonitrile in the presence of thio-CDI [35]. Finally, they devised an excellent protocol for the cyclization of 1,2-amino alcohol-based thiourea derivatives to yield 2-phenylaminothiazolines under mild conditions (rt, THF, CDI or thio-CDI).

**Figure 2:**
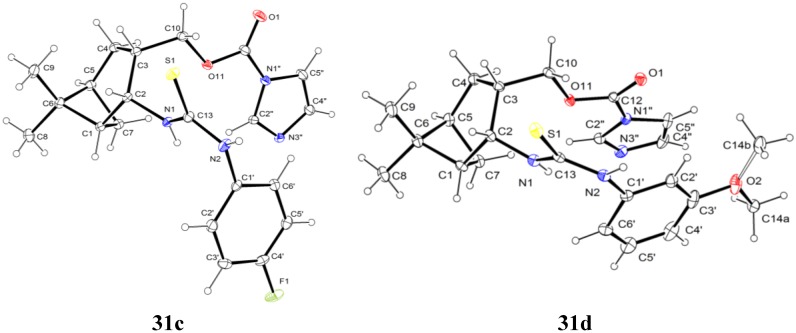
ORTEP plots of the configurations of the major diastereoisomers **31c** and **31d** (the C atom of the methoxy group (C14) is disordered in a 1:1 ratio).

When we applied the above-mentioned mild conditions, we observed the formation of *O*-imidazolylcarbonyl intermediates **29**–**33** (similar intermediates were presumed, but could not be isolated in Bernacki’s work) as single products even when the reaction mixture was subjected to conventional heating from room temperature to reflux (Table 1). The structures of intermediates **31c** and **31d** were determined by X-ray crystallography (Figure 2) and NMR measurements.

However, when microwave irradiation was applied to the isolated intermediates **29**–**33** in THF, we obtained the desired 2-imino-1,3-thiazines **25**–**28** as main products in acceptable yields in a short time (60 min) (Scheme 3, Table 2).

**Table 2:** 2-Phenylimono-1,3-thiazines **24**–**28**.

General Structure	R^1^	R^2^	R^3^	Compound No.
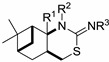	Me	H	Ph	**24a**
	H	H	Ph	**26a**
	H	H	4-MeC_6_H_4_	**26b**
	H	H	4-FC_6_H_4_	**26c**
	H	H	3-MeOC_6_H_4_	**26d**
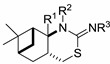	H	H	Ph	**27a**
	H	CH_2_Ph	Ph	**28a**
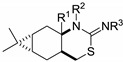	Me	H	Ph	**25a**

The ring-closure process could be carried out in one step by applying CDI in THF under microwave conditions. Moreover, we observed that 2-thioxo-1,3-oxazines **34**–**37** were also formed as minor products in 10%–24% yields. The transformation of **20a** was an exception, when thiazine **25a** was isolated as a single product. Besides NMR assignments, the structures of **35** and **36** were proved by independent synthesis starting from the corresponding amino alcohols and thiophosgene.

**Scheme 2:**
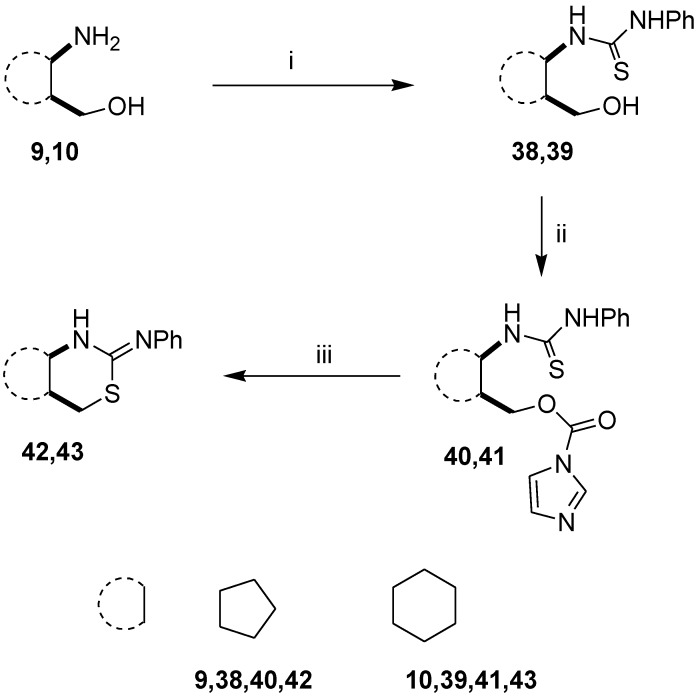
Extension of the ring-closure process.

The above procedure was subsequently extended to 1-(2-hydroxymethylcycloalkyl)thioureas. Starting from cycloalkane-based 1,3-amino alcohols **9** and **10**, thioureas **38** and **39** were prepared by a literature method [33]. When **38** and **39** were treated with CDI in THF, rapid conversion to intermediates **40** and **41** was observed and in this case the ring-closure proceeded under conventional heating to yield 1,3-thiazines **42** and **43** (Scheme 2). We observed that the developed method could be applied more easily in the case of thioureas with sterically less hindered structures.

*Reagents and conditions*: (i) 1.05 equiv. PhNCS, toluene, rt, 4 h; (ii) 1.5 equiv. CDI, THF, rt, 1 h; (iii) THF, reflux, 3 h, yield: 73%–86%

### 2.3. Antiproliferative Activities

**Scheme 3:**
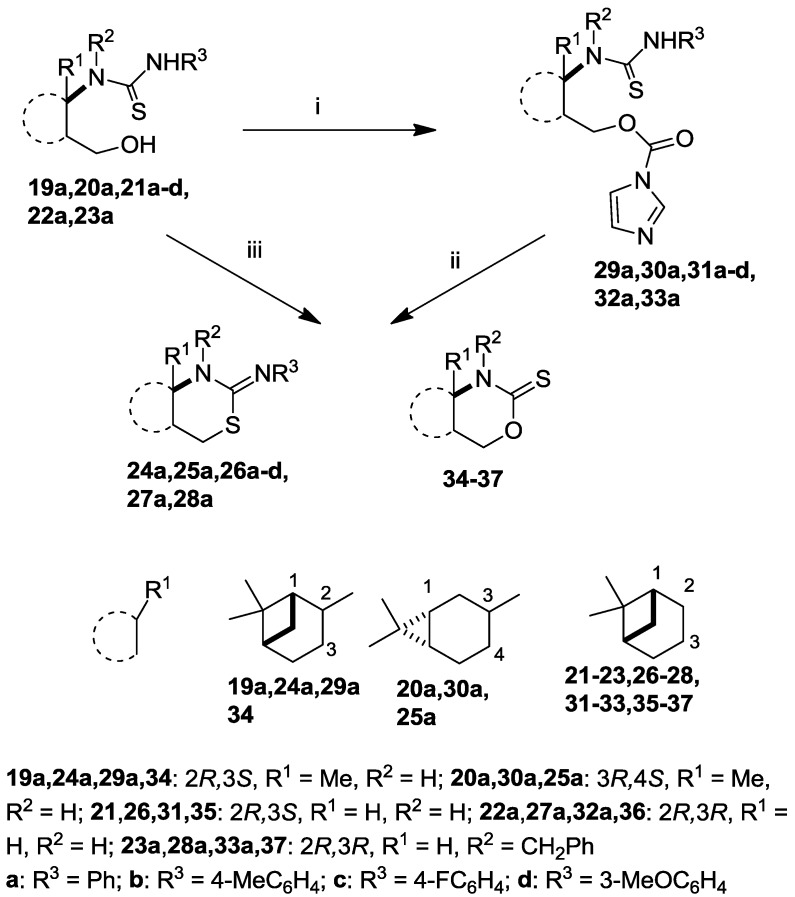
CDI mediated ring closure of thioureas **19**–**23**.

The novel 2-imino-1,3-thiazines **24**–**28** (Scheme 3) and some of their analogues were subjected to *in vitro* pharmacological studies in order to investigate their antiproliferative properties on a panel of human adherent cancer cell lines. The results of MTT assays are presented in Table 3. Carane-based compound **25a** proved to be the most potent of the tested compounds, exhibiting a cell growth-inhibiting capacity comparable to that of the reference agent cisplatin. Compounds with a pinane ring were generally less potent, while the introduction of a methyl group at position 2 (Scheme 3) favoured the action (**24a**). Substitution of the *N*-phenyl ring had a limited and inconsequential impact on the efficacy (**26a**–**d**), while the introduction of a *N*-benzyl function onto the 1,3-thiazine skeleton was disadvantageous (**28a**). Since no substantial difference was observed between the effects of **26a** and **27a**, the configuration of C-3 (*cis* or *trans* ring fusion, Scheme 3) also seems irrelevant. On the other hand, the 2-thioxo-1,3-oxazine analogue (**35**) was completely ineffective, indicating that the arylimino substituent is an essential part of the molecule. Replacement of the pinane or carane ring system with cyclopentane (**42**) or cyclohexane (**43**) also led to ineffective congeners, demonstrating the crucial role of the bicyclic monoterpene as a building block for the design and synthesis of novel antiproliferative agents.

*Reagents and conditions*: (i) 1.5 equiv. CDI, THF, rt, 6 h, yield: 52%–78%; (ii) MW, 200 W, 125 °C, 1 h, THF, yield: 49%–67% for **24**–**28** and 8–22% for **34**–**37**; (iii) 1.5 equiv. CDI, MW, 200 W, 125 °C, 1 h, yield: 47%–64% for **24**–**28** and 10%–24% for **34**–**37**.

**Table 3:** Antiproliferative effects of 2-imino-1,3-thiazines **24**–**28**, **42**, **43** and 2-thioxo-1,3-oxazine **35** on human cancer cell lines.

Compnd.	Conc.		Growth Inhibition, % ± SEM ^a^							
			HeLa		A2780		MCF7		A431	
**24a**	10 µM		-		67.45 ± 0.83		39.40 ± 2.41		73.01 ± 1.52	
	30 µM		25.59 ± 1.93		86.02 ± 0.27		62.08 ± 1.78		83.15 ± 1.25	
**25a**	10 µM		-		65.24 ± 1.65		65.91 ± 0.96		60.54 ± 1.79	
	30 µM		96.40 ± 0.28		96.42 ± 0.15		86.09 ± 1.20		94.17 ± 0.51	
**26a**	10 µM		-		-		26.52 ± 2.79		67.70 ± 1.49	
	30 µM		22.03 ± 1.57		68.93 ± 0.97		50.45 + 1.79		85.68 ± 0.74	
**26b**	10 µM		-		45.45 ± 2.01		-		65.20 ± 1.47	
	30 µM		37.02 ± 2.20		58.67 ± 1.29		43.66 ± 2.32		74.49 ± 1.02	
**26c**	10 µM		44.90 ± 0.88		40.02 ± 0.88		21.55 ± 0.99		32.93 ± 1.20	
	30 µM		53.60 ± 1.07		56.86 ± 1.17		30.93 ± 1.29		34.20 ± 1.02	
**26d**	10 µM		27.40 ± 0.52		20.87 ± 1.87		-		34.92 ± 2.66	
	30 µM		33.67 ± 2.61		92.17 ± 0.51		63.84 ± 2.36		82.46 ± 1.11	
**27a**	10 µM		-		37.26 ± 2.35		43.83 ± 0.99		77.32 ± 0.94	
	30 µM		21.57 ± 0.92		42.80 ± 2.78		47.15 ± 2.93		80.05 ± 1.15	
**28a**	10 µM		46.83 ± 1.42		23.10 ± 1.00		-		-	
	30 µM		42.24 ± 2.48		50.23 ± 0.52		23.84 ± 1.08		-	
**35**	10 µM		-		-		-		-	
	30 µM		-		-		-		-	
**42**	10 µM		-		-		-		-	
	30 µM		-		41.23 ± 1.30		-		-	
**43**	10 µM		-		-		-		-	
	30 µM		24.07 ± 2.96		-		32.59 ± 1.49		-	
Cisplatin	10 µM		42.61 ± 2.33		83.57 ± 1.21		53.03 ± 2.29		88.54 ± 0.50	
	30 µM		99.93 ± 0.26		95.02 ± 0.28		86.90 ± 1.24		90.18 ± 1.78	

^a^ Substances eliciting less than 20% inhibition of cell proliferation were regarded as ineffective and the results are not presented.

## 3. Experimental Section

### 3.1. General Information

^1^H- and ^13^C-NMR spectra were recorded on a Bruker Avance DRX 400 spectrometer (400 MHz, δ = 0 (TMS)), in an appropriate solvent. Chemical shifts are expressed in ppm (δ) relative to TMS as internal reference. *J* values are given in Hz. FT-IR spectra were recorded on a Perkin-Elmer Spectrum 100 instrument. Microanalyses were performed on a Perkin-Elmer 2400 elemental analyser. Microwave reactions were carried out by heating at 125 °C and 200 W for 1 h in a CEM Discover LabMate microwave reactor. Optical rotations were obtained with a Perkin-Elmer 341 polarimeter. Melting points were determined on a Kofler apparatus and are uncorrected. Chromatographic separations were carried out on Merck Kieselgel 60 (230–400 mesh ASTM). Reactions were monitored with Merck Kieselgel 60 F254-precoated TLC plates (0.25 mm thickness). All the chemicals and solvents were used as supplied.

Compounds **5**–**18** and thioureas **19a**, **20a**, **38** and **39** were prepared by literature methods; all their spectroscopic data and physical properties were similar to those reported therein [9,13,27,28,29,31,33].

### 3.2. Synthesis

#### 3.2.1. General Procedure for the Synthesis of Thioureas **21**–**23**

Amino alcohol **16**, **17** or **18** (1.08 mmol) and the appropriate isothiocyanate (1.14 mmol) were dissolved in toluene (80 mL) and the mixture was stirred at room temperature for 6 h, except that in the case of *N*-benzylamino alcohol **18**, heating at 50 °C for 6 h was indicated. The resulting mixtures were then evaporated to dryness, filtered and washed with *n*-hexane. The purities of the products obtained were determined via NMR to be >97%.

*(1R,2R,3S,5R)-1-(3-Hydroxymethyl-6,6-dimethylbicyclo[3.1.1]hept-2-yl)-3-phenylthiourea* (**21a**): 0.31 g (95%); mp 152–155 °C, 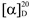 = +52.0 (*c* 0.25, MeOH), 1H-NMR (CDCl_3_) δ (ppm) 0.94 (1H, d, *J* = 9.9 Hz), 1.01 (3H, s), 1.23 (3H, s), 1.63–1.70 (1H, m), 1.72–1.79 (1H, m), 1.88–1.94 (1H, m), 1.98–2.18 (3H, m), 2.54–2.65 (1H, m), 3.48-3.63 (2H, m), 5.15 (1H, *t*, *J* = 8.2 Hz), 7.16 (1H, d, *J* = 8.5 Hz), 7.19-7.44 (5H, m), 7.68 (1H, s). ^13^C-NMR (CDCl_3_) δ (ppm) 21.1, 26.5, 26.6, 26.8, 30.0, 32.6, 39.3, 40.7, 46.4, 57.0, 65.1, 125.7, 127.6, 130.3, 136.5, 180.2. Anal. Calcd for C_17_H_24_N_2_OS (304.45): C, 67.07; H, 7.95; N, 9.20; S, 10.53%; Found: C, 67.39; H, 8.13; N, 9.01; S, 10.42%.

*(1R,2R,3S,5R)-1-(4-Tolyl)-3-(3-hydroxymethyl-6,6-dimethylbicyclo[3.1.1]hept-2-yl)thiourea* (**21b**): 0.32 g (92%); mp 98–101 °C, 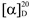 = +63.0 (*c* 0.25, MeOH), 1H-NMR (CDCl_3_) δ (ppm) 0.92 (1H, d, *J* = 10.1 Hz), 1.01 (3H, s), 1.22 (3H, s), 1.62–1.68 (1H, m), 1.73–1.86 (1H, m), 1.87–1.93 (1H, m), 1.98–2.16 (3H, m), 2.35 (3H, s), 2.54–2.64 (1H, m), 3.47–3.61 (2H, m), 5.15 (1H, br s), 7.05 (1H, d, *J* = 8.5 Hz), 7.09 (2H, d, *J* = 8.2 Hz), 7.20 (2H, d, *J* = 8.2 Hz). ^13^C-NMR (CDCl_3_) δ (ppm) 21.2, 21.5, 26.8, 26.9, 30.0, 32.8, 39.4, 40.8, 46.5, 57.0, 65.3, 126.0, 131.0, 132.8, 137.7, 151.9, 180.1. Anal. Calcd for C_18_H_26_N_2_OS (318.48): C, 67.88; H, 8.23; N, 8.80; S, 10.07%; Found: C, 68.13; H, 8.35; N, 8.69; S, 9.94%.

*(1R,2R,3S,5R)-1-(4-Fluorophenyl)-3-(3-hydroxymethyl-6,6-dimethylbicyclo[3.1.1]hept-2-yl)thiourea* (**21c**): 0.32 g (93%); mp 162–163 °C, 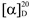 = +55.0 (*c* 0.25, MeOH), ^1^H-NMR (CDCl_3_) δ (ppm) 0.97 (1H, d, *J* = 9.8 Hz), 1.00 (3H, s), 1.22 (3H, s), 1.62–1.72 (1H, m), 1.87–1.94 (1H, m), 1.98–2.16 (4H, m), 2.51–2.63 (1H, m), 3.51 (1H, dd, *J* = 4.6, 10.5 Hz), 3.61 (1H, dd, *J* = 3.2, 10.7 Hz), 5.11 (1H, br s), 7.01–7.26 (4H, m). ^13^C-NMR (CDCl_3_) δ (ppm) 21.1, 26.6, 26.7, 29.9, 32.4, 39.3, 40.7, 46.3, 57.0, 65.0, 117.1 (d, *J* = 23.7 Hz), 128.1 (d, *J* = 8.5 Hz), 162.6, 180.1. Anal. Calcd for C_17_H_23_FN_2_OS (322.44): C, 63.32; H, 7.19; N, 8.69; S, 9.94%; Found: C, 63.49; H, 7.29; N, 8.48; S, 9.77%.

*(1R,2R,3S,5R)-1-(3-Methoxyphenyl)-3-(3-hydroxymethyl-6,6-dimethylbicyclo[3.1.1]hept-2-yl)thiourea* (**21d**): 0.33 g (90%); mp 118–120 °C, 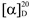 = +57.0 (*c* 0.20, MeOH), ^1^H-NMR (CDCl_3_) δ (ppm) 0.99 (1H, d, *J* = 8.8 Hz), 1.01 (3H, s), 1.23 (3H, s), 1.65–1.74 (1H, m), 1.78–1.92 (2H, m), 1.99–2.19 (3H, m), 2.55–2.66 (1H, m), 3.53 (1H, dd, *J* = 4.5, 10.7 Hz), 3.61 (1H, br d, *J* = 10.5 Hz), 3.79 (3H, s), 5.17 (1H, br s), 6.72–6.83 (3H, m), ), 7.23–7.34 (2H, m). ^13^C-NMR (CDCl_3_) δ (ppm) 21.1, 26.7, 26.8, 29.9, 32.6, 39.3, 40.8, 46.4, 55.8, 57.1, 65.1, 110.9, 113.1, 117.3, 128.8, 131.1, 137.6, 161.1, 179.9. Anal. Calcd for C_18_H_26_N_2_O_2_S (334.48): C, 64.64; H, 7.84; N, 8.39; S, 9.59%; Found: C, 64.73; H, 7.99; N, 8.11; S, 9.38%.

*(1R,2R,3R,5R)-1-(3-Hydroxymethyl-6,6-dimethylbicyclo[3.1.1]hept-2-yl)-3-phenylthiourea* (**22a**): 0.31 g (94%); mp 151–155 °C, 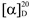 = −19.0 (*c* 0.25, MeOH), ^1^H-NMR (CDCl_3_) δ (ppm) 0.94 (3H, s), 1.23 (3H, s), 1.30 (1H, d, *J* = 10.2 Hz), 1.57–1.64 (1H, m), 1.71–1.88 (2H, m), 1.92–2.00 (1H, m), 2.06–2.13 (1H, m), 3.42–3.50 (1H, m), 3.66–3.83 (2H, m), 4.91 (1H, t, *J* = 7.7 Hz), 6.16 (1H, d, *J* = 9.4 Hz), 7.18 (2H, d, *J* = 7.7 Hz), 7.29 (1H, t, *J* = 7.0 Hz), 7.43 (2H, t, *J* = 7.5 Hz), 7.82 (1H, s). ^13^C-NMR (CDCl_3_) δ (ppm) 19.8, 23.8, 27.1, 27.3, 39.3, 40.4, 41.1, 46.6, 56.6, 64.4, 125.3, 127.7, 130.7, 135.8, 159.6, 179.5. Anal. Calcd for C_17_H_24_N_2_OS (304.45): C, 67.07; H, 7.95; N, 9.20; S, 10.53%; Found: C, 67.31; H, 7.80; N, 9.30; S, 10.41%.

*(1R,2R,3R,5R)-1-Benzyl-1-(3-hydroxymethyl-6,6-dimethylbicyclo[3.1.1]hept-2-yl)-3-phenylthiourea* (**23a**): 0.36 g (85%); mp 177–178 °C, 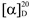 = +7.0 (*c* 0.25, MeOH), ^1^H-NMR (CDCl_3_) δ (ppm) 1.07 (3H, s), 1.26 (3H, s), 1.65 (1H, d, *J* = 10.2 Hz), 1.75–1.86 (1H, m), 1.89–2.01 (3H, m), 2.09–2.15 (1H, m), 2.23–2.31 (1H, m), 3.55–3.63 (1H, m), 3.92–3.99 (1H, m), 4.73–4.90 (2H, m), 6.01 (1H, br s), 7.03–7.45 (10H, m). ^13^C-NMR (CDCl_3_) δ (ppm) 19.8, 25.6, 27.3, 27.5, 35.6, 40.0, 42.8, 45.8, 49.0, 59.6, 62.1, 126.4, 126.5, 126.6, 128.5, 128.9, 129.7, 139.3, 139.8, 183.1. Anal. Calcd for C_24_H_30_N_2_OS (394.57): C, 73.06; H, 7.66; N, 7.10; S, 8.13%; Found: C, 73.40; H, 7.86; N, 7.05; S, 8.38%.

#### 3.2.2. General Procedure for the Reactions of Thioureas **19**–**23** with CDI to Yield Intermediates **29**–**33**

To the respective thiourea (1.25 mmol) **19**–**23**, **38** or **39** in THF solution (12 mL), CDI (0.306 g, 1.88 mmol) was added at room temperature. The reaction was stirred for 2–6 h at room temperature (TLC monitoring), followed by careful evaporation of the solvent at 35 °C. The crude products obtained were purified by flash column chromatography on silica gel.

*(1R,2R,3S,5R)-[2,6,6-Trimethyl-2-(3-phenylthioureido)bicyclo[3.1.1]hept-3-yl]methyl imidazole-1-carboxylate* (**29a**): 0.27 g (52%); oil, 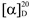 = +32.0 (*c* 0.25, MeOH), ^1^H-NMR (CDCl_3_) δ (ppm) 0.92 (3H, s), 1.07 (1H, d, *J* = 10.1 Hz), 1.22 (3H, s), 1.42 (3H, s), 1.60–1.76 (1H, m), 1.92–2.20 (3H, m), 2.90–3.00 (1H, m), 4.35 (2H, d, *J* = 5.2 Hz), 5.26 (1H, t, *J* = 9.4 Hz), 6.25 (1H, d, *J* = 9.0 Hz), 7.05–7.39 (7H, m), 7.80 (1H, br s), 7.92 (1H, s). ^13^C-NMR (CDCl_3_) δ (ppm) 21.3, 22.5, 26.7, 26.6, 29.6, 30.1, 39.5, 40.8, 46.5, 55.6, 71.5, 117.5, 125.3, 128.5, 130.0, 130.4, 131.2, 135.5, 148.7, 181.5. Anal. Calcd for C_22_H_28_N_4_O_2_S (412.55): C, 64.05; H, 6.84; N, 13.58; S, 7.77%; Found: C, 63.89; H, 6.91; N, 13.73; S, 7.65%.

*(1R,2R,3S,5R)-[2,6,6-Trimethyl-2-(3-phenylthioureido)bicyclo[3.1.1]hept-3-yl]methyl imidazole-1-carboxylate* (**30a**): 0.32 g (62%); oil, 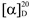 = –140.0 (*c* 0.25, MeOH), ^1^H-NMR (CDCl_3_) δ (ppm) 0.58 (1H, t, *J* = 8.5 Hz), 0.76–0.85 (2H, m), 0.93 (3H, s), 1.05 (3H, s), 1.28–1.55 (3H, m), 1.67 (3H, s), 1.72–1.82 (1H, m), 3.36 (1H, dd, *J* = 2.7, 11.1 Hz), 3.75 (1H,dd, *J* = 7.1, 14.0 Hz), 3.84 (1H, dd, *J* = 11.3, 16.7 Hz), 3.95 (1H, dd, *J* = 2.7, 11.2 Hz), 7.19 (1H, br s), 7.22–7.46 (7H, m). ^13^LC-NMR (CDCl_3_) δ (ppm) 15.6, 19.1, 19.3, 24.0, 29.0, 30.1, 31.1, 45.4, 63.0, 69.1, 122.5, 126.6, 126.9, 127.6, 128.8, 130.1, 137.0, 143.0, 180.0. Anal. Calcd for C_22_H_28_N_4_O_2_S (412.55): C, 64.05; H, 6.84; N, 13.58; S, 7.77%; Found: C, 64.21; H, 6.97; N, 13.23; S, 7.71%.

*(1R,2R,3S,5R)-[6,6-Dimethyl-2-(3-phenylthioureido)bicyclo[3.1.1]hept-3-yl]methyl imidazole-1-carboxylate* (**31a**): 0.39 g (78%); oil, 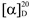 = +39.0 (*c* 0.32, MeOH), ^1^H-NMR (CDCl_3_) δ (ppm) 0.86 (1H, d, *J* = 10.3 Hz), 1.03 (3H, s), 1.26 (3H, s), 1.65–1.72 (1H, m), 1.95–2.26 (4H, m), 2.90–3.00 (1H, m), 4.39 (2H, d, *J* = 5.4 Hz), 5.29 (1H, t, *J* = 9.2 Hz), 6.26 (1H, d, *J* = 9.0 Hz), 7.00–7.30 (7H, m), 7.85 (1H, br s), 7.92 (1H, s). ^13^C-NMR (CDCl_3_) δ (ppm) 21.1, 26.5, 26.6, 29.6, 30.1, 39.5, 40.7, 46.5, 55.8, 71.5, 117.3, 125.5, 128.1, 130.1, 130.5, 131.0, 135.9, 148.9, 181.0. Anal. Calcd for C_21_H_26_N_4_O_2_S (398.52): C, 63.29; H, 6.58; N, 14.06; S, 8.05%; Found: C, 63.54; H, 6.60; N, 13.84; S, 7.91%.

*(1R,2R,3S,5R)-{6,6-Dimethyl-2-[3-(4-tolyl)thioureido]bicyclo[3.1.1]hept-3-yl}methyl imidazole-1-carboxylate* (**31b**): 0.36 g (69%); mp 148–150 °C, 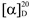 = +81.0 (*c* 0.265, MeOH), ^1^H-NMR (CDCl_3_) δ (ppm) 0.97 (3H, s), 1.19 (1H, d, *J* = 10.0 Hz), 1.24 (3H, s), 1.66–1.75 (1H, m), 1.91–1.99 (2H, m), 2.06–2.16 (1H, m), 2.19–2.30 (1H, m), 2.24 (3H, s), 2.79–2.91 (1H, m), 4.34–4.44 (2H, m), 5.14 (1H, t, *J* = 8.8 Hz), 7.01–7.09 (3H, m), 7.25 (1H, d, *J* = 8.2 Hz), 7.57 (1H, s), 7.72 (1H, d, *J* = 8.9 Hz), 8.23 (1H, s), 9.28 (1H, s). ^13^C-NMR (DMSO–*d*_6_) δ (ppm) 21.3, 21.5, 27.1, 27.2, 29.9, 30.4, 39.3, 47.0, 53.7, 59.0, 71.8, 118.4, 124.1, 129.7, 131.0, 134.2, 137.2, 137.7, 149.3, 181.6. Anal. Calcd for C_22_H_28_N_4_O_2_S (412.55): C, 64.05; H, 6.84; N, 13.58; S, 7.77%; Found: C, 64.39; H, 6.93; N, 13.27; S, 7.56%.

*(1R,2R,3S,5R)-{6,6-Dimethyl-2-[3-(4-fluorophenyl)thioureido]bicyclo[3.1.1]hept-3-yl}methyl imidazole-1-carboxylate* (**31c**): 0.39 g (75%); mp 201–203 °C, 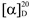 = +44.0 (*c* 0.35, MeOH), ^1^H-NMR (CDCl_3_) δ (ppm) 0.87 (1H, d, *J* = 10.5 Hz), 1.03 (3H, s), 1.24 (3H, s), 1.65–1.72 (1H, m), 1.96–2.26 (4H, m), 2.91–3.01 (1H, m), 4.37–4.46 (2H, m), 5.26 (1H, t, *J* = 9.9 Hz), 6.20 (1H, br s), 6.94–7.15 (5H, m), 7.30 (1H, s), 7.78 (1H, br s), 8.14 (1H, br s). ^13^C-NMR (DMSO–*d*_6_) δ (ppm) 21.0, 26.5, 26.6, 29.7, 30.1, 39.4, 40.6, 46.6, 55.4, 71.6, 117.2, 117.3, 117.4, 127.7, 127.8, 148.8, 160.4, 162.9, 181.3. Anal. Calcd for C_21_H_25_FN_4_O_2_S (416.51): C, 60.56; H, 6.05; N, 13.45; S, 7.70%; Found: C, 60.79; H, 6.41; N, 13.08; S, 7.53%.

*(1R,2R,3S,5R)-{6,6-Dimethyl-2-[3-(3-methoxyphenyl)thioureido]bicyclo[3.1.1]hept-3-yl}methyl imidazole-1-carboxylate* (**31d**): 0.39 g (73%); mp 128–130 °C, 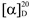 = +9.0 (*c* 0.26, MeOH), ^1^H-NMR (CDCl_3_) δ (ppm) 0.91 (1H, d, *J* = 10.6 Hz), 1.03 (3H, s), 1.26 (3H, s), 1.66–1.72 (1H, m), 1.97–2.02 (1H, m), 2.06–2.28 (3H, m), 2.90–3.00 (1H, m), 3.73 (3H, s), 4.34–4.44 (2H, m), 5.30 (1H, t, *J* = 9.8 Hz), 6.41 (1H, d, *J* = 9.7 Hz), 6.60–6.69 (3H, m), 7.02 (1H, s), 7.17 (1H, t, *J* = 8.1 Hz), 7.22 (1H, s), 7.79 (1H, s), 7.92 (1H, s). ^13^C-NMR (CDCl_3_) δ (ppm) 21.2, 26.6, 26.7, 29.6, 30.2, 39.6, 40.7, 46.6, 55.8, 55.9, 71.5, 110.6, 111.2, 113.4, 117.1, 117.3, 131.0, 131.3, 137.0, 148.9, 161.4, 180.8. Anal. Calcd for C_22_H_28_N_4_O_3_S (428.55): C, 61.66; H, 6.59; N, 13.07; S, 7.48%; Found: C, 61.83; H, 6.84; N, 12.89; S, 7.53%.

*(1R,2R,3R,5R)-[6,6-Dimethyl-2-(3-phenylthioureido)bicyclo[3.1.1]hept-3-yl]methyl imidazole-1-carboxylate* (**32a**): 0.33 g (67%); oil, 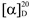 = −15.0 (*c* 0.30, MeOH), ^1^H-NMR (CDCl_3_) δ (ppm) 0.95 (3H, s), 1.26 (3H, s), 1.31 (1H, d, *J* = 9.5 Hz), 1.61–1.72 (1H, m), 1.94–2.19 (5H, m), 4.54 (1H, dd, *J* = 6.6, 10.8 Hz), 4.65 (1H, dd, *J* = 6.6, 10.7 Hz), 5.04 (1H, t, *J* = 8.7 Hz), 6.16 (1H, d, *J* = 9.2 Hz), 7.06 (1H, s), 7.13 (2H, d, *J* = 7.8 Hz), 7.24–7.29 (1H, m), 7.40 (2H, t, *J* = 7.9 Hz), 7.46 (1H, br s), 8.13 (1H, br s), 8.16 (1H, s). ^13^C-NMR (CDCl_3_) δ (ppm) 19.9, 23.6, 26.7, 27.5, 35.6, 40.2, 40.646.2, 55.7, 70.4, 117.6, 125.2, 127.5, 130.6, 131.0, 136.5, 149.2, 180.3. Anal. Calcd for C_21_H_26_N_4_O_2_S (398.52): C, 63.29; H, 6.58; N, 14.06; S, 8.05%; Found: C, 63.43; H, 6.75; N, 13.85; S, 8.16%.

*(1R,2R,3R,5R)-[6,6-Dimethyl-2-(1-benzyl-3-phenylthioureido)bicyclo[3.1.1]hept-3-yl]methyl imidazole-1-carboxylate* (**33a**): 0.37 g (60%); oil, 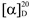 = −15.0 (*c* 0.30, MeOH), ^1^H-NMR (CDCl_3_) δ (ppm) 1.09 (3H, s), 1.32 (3H, s), 1.68 (1H, d, *J* = 10.1 Hz), 1.80–1.89 (1H, m), 1.95–2.11 (2H, m), 2.20 (1H, t, *J* = 5.4 Hz), 2.33–2.50 (2H, m), 4.65 (1H, dd, *J* = 7.3, 11.6 Hz), 4.80–4.89 (3H, m), 6.50 (1H, br d, *J* = 10.6 Hz), 7.03–7.55 (12H, m), 8.20 (1H, s). ^13^C-NMR (CDCl_3_) δ (ppm) 20.1, 25.7, 27.1, 28.1, 33.3, 39.9, 42.5, 45.8, 49.1, 58.9, 70.3, 117.7, 126.3, 126.5, 128.7, 128.9, 129.2, 130.9, 131.8, 136.1, 137.1, 140.0, 149.2, 184.2. Anal. Calcd for C_28_H_32_N_4_O_2_S (488.22): C, 68.82; H, 6.60; N, 11.47; S, 6.56%; Found: C, 68.63; H, 6.49; N, 11.72; S, 6.83%.

*2-[(3-Phenylthioureido)cyclopent-3-yl]methyl imidazole-1-carboxylate* (**40**): 0.28 g (65%); oil, ^1^H-NMR (CDCl_3_) δ (ppm) 1.36–1.49 (2H, m), 1.54–1.75 (2H, m), 1.92–2.02 (1H, m), 2.08–2.18 (1H, m), 2.73–2.84 (1H, m), 4.31 (1H, dd, *J* = 5.3, 11.0 HZ), 4.39 (1H, dd, *J* = 6.1, 11.1 Hz), 4.96–5.04 (1H, m), 5.96 (1H, d, *J* = 7.9 Hz), 7.03 (1H, s), 7.10–7.15 (2H, m), 7.19–7.25 (1H, m), 7.30–7.37 (3H, m), 7.99 (1H, br s), 8.03 (1H, s). ^13^C-NMR (CDCl_3_) δ (ppm) 22.5, 27.7, 33.0, 39.9, 57.9, 69.0, 117.5, 125.6, 128.1, 130.7, 131.1, 136.2, 137.5, 148.9, 181.3. Anal. Calcd for C_17_H_20_N_4_O_2_S (344.43): C, 59.28; H, 5.85; N, 16.27; S, 9.39%; Found: C, 59.28; H, 5.85; N, 16.27; S, 9.39%.

*2-[(3-Phenylthioureido)cyclohex-3-yl]methyl imidazole-1-carboxylate* (**41**): 0.26 g (57%); oil, ^1^H-NMR (CDCl_3_) δ (ppm) 0.86–1.00 (1H, m), 1.06–1.20 (1H, m), 1.32–1.45 (1H, m), 1.59–1.79 (4H, m), 1.86–1.97 (1H, m), 2.24–2.36 (1H, m), 4.23–4.35 (2H, m), 4.96–5.04 (1H, m), 6.23 (1H, d, *J* = 9.0 Hz), 7.06–7.09 (1H, m), 7.23–7.28 (2H, m), 7.37 (1H, t, *J* = 7.5 Hz), 7.47–7.53 (3H, m), 8.20 (1H, s). ^13^C-NMR (CDCl_3_) δ (ppm) 21.0, 24.7, 26.4, 32.2, 32.6, 34.3, 52.5, 122.8, 123.5, 125.6, 125.7, 129.3, 135.0, 146.1, 156.4, 171.2. Anal. Calcd for C_18_H_22_N_4_O_2_S (358.46): C, 60.31; H, 6.19; N, 15.63; S, 8.95%; Found: C, 60.47; H, 6.23; N, 15.12; S, 8.69%.

#### 3.2.3. General Procedure for the Synthesis of 2-Imino-1,3-thiazines **24**–**28** and 2-Thioxo-1,3-oxazines **34**–**37**

*Method A*: A CEM Discover 10 mL vial containing the respective thiourea (1.25 mmol) **19**–**23** in THF solution (12 mL) and CDI (0.306 g, 1.88 mmol) and sealed with a Teflon cap was irradiated at 125 °C (200 W) for 60 min with a ramp time of 5 min. Next, the solvent was evaporated off under reduced pressure and the residue was purified by flash column chromatography on silica gel; elution with *n*-hexane/EtOAc (4:1) gave 2-imino-1,3-thiazines and 2-thioxo-1,3-oxazines.

*Method B*: A CEM Discover 10 mL vial containing the respective intermediates (1.25 mmol) **29**–**33** in THF solution (12 mL) and sealed with a Teflon cap was irradiated at 125 °C (200 W) for 60 min with a ramp time of 5 min. Next, the solvent was evaporated off under reduced pressure and the residue was purified by flash column chromatography on silica gel; elution with *n*-hexane/EtOAc (4:1) gave 2-imino-1,3-thiazines and 2-thioxo-1,3-oxazines.

*(1R,2R,7S,9R)-(10,10-Dimethyl-5-thia-3-azatricyclo[7.1.1.0^2,7^]undec-4-ylidene)phenylamine* (**26a**): *Method A*: 0.197 g (55%), *method B*: 0.204 g (57%); mp 124–128 °C, 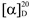 = +142.0 (*c* 0.25, MeOH), ^1^H-NMR (CDCl_3_) δ (ppm) 0.95 (3H, s), 1.24 (3H, s), 1.30 (1H, d, *J* = 10.5 Hz), 1.50–1.58 (1H, m), 1.92–1.99 (2H, m), 2.13–2.30 (2H, m), 2.61–2.73 (2H, m), 2.87 (1H, t, *J* = 13.4 Hz), 3.92 (1H, t, *J* = 8.9 Hz), 6.88–7.30 (5H, m). ^13^C-NMR (CDCl_3_) δ (ppm) 20.9, 26.9, 27.0, 33.1, 34.1, 35.7, 39.0, 41.1, 47.7, 54.9, 122.9, 123.7, 129.3, 137.7, 160.4. Anal. Calcd for C_17_H_22_N_2_S (286.43): C, 71.28; H, 7.74; N, 9.78; S, 11.19%; Found: C, 71.39; H, 8.00; N, 9.21; S, 11.11%.

*(1R,2R,7S,9R)-10,10-Dimethyl-5-oxa-3-azatricyclo[7.1.1.0^2,7^]undecane-4-thione* (**35**): *Method A*: 0.053 g (20%), *method B*: 0.058 g (22%); this compound was also isolated from transformations of thioureas **26c**–**d**, in 18%–24% yield; mp 160–161 °C, 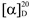 = +112.0 (*c* 0.25, MeOH), ^1^H-NMR (CDCl_3_) δ (ppm) 0.91 (3H, s), 1.26 (3H, s), 1.28 (1H, d, *J* = 11.8 Hz), 1.45–1.52 (1H, m), 1.97–2.24 (4H, m), 2.64–2.75 (1H, m), 3.85–3.88 (1H, m), 3.91 (1H, dd, *J* = 10.2, 21.1 Hz), 4.24 (1H, dd, *J* = 6.1, 10.8 Hz), 7.53 (1H, br s). ^13^C-NMR (CDCl_3_) δ (ppm) 20.4, 25.3, 26.6, 26.7, 28.0, 39.6, 40.5, 46.1, 54.2, 72.6, 192.5. Anal. Calcd for C_11_H_17_NOS (211.32): C, 62.52; H, 8.11; N, 6.63; S, 15.17%; Found: C, 62.77; H, 8.36; N, 6.41; S, 15.00%.

*(1R,2R,7S,9R)-(10,10-Dimethyl-5-thia-3-azatricyclo[7.1.1.0^2,7^]undec-4-ylidene)-p-tolylamine* (**26b**): *Method A*: 0.199 g (53%), *method B*: 0.203 g (54%); mp 199–203 °C, 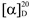 = +170.0 (*c* 0.25, MeOH), ^1^H-NMR (CDCl_3_) δ (ppm) 0.94 (3H, s), 1.23 (3H, s), 1.30 (1H, d, *J* = 10.6 Hz), 1.49–1.57 (1H, m), 1.88–1.99 (2H, m), 2.11–2.29 (2H, m), 2.31 (3H, s), 2.59–2.71 (2H, m), 2.87 (1H, t, *J* = 13.4 Hz), 3.91 (1H, d, *J* = 8.3 Hz), 5.01 (1H, br s), 6.80 (1H, d, *J* = 8.1 Hz), 7.08 (1H, d, *J* = 8.1 Hz). ^13^C-NMR (CDCl_3_) δ (ppm) 20.9, 21.3, 26.9, 27.0, 34.2, 35.8, 39.1, 41.2, 47.8, 54.8, 122.5, 129.9, 133.0, 146.2, 159.7. Anal. Calcd for C_18_H_24_N_2_S (300.46): C, 71.95; H, 8.05; N, 9.32; S, 10.67%; Found: C, 71.99; H, 8.21; N, 9.10; S, 10.70%.

*(1R,2R,7S,9R)-(10,10-Dimethyl-5-thia-3-azatricyclo[7.1.1.0^2,7^]undec-4-ylidene)-3-Methoxyphenylamine* (**26c**): *Method A*: 0.225 g (57%), *method B*: 0.233 g (59%); mp 154–155 °C, 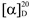 = +234.0 (*c* 0.25, MeOH), ^1^H-NMR (CDCl_3_) δ (ppm) 0.95 (3H, s), 1.25 (3H, s), 1.30 (1H, d, *J* = 10.6 Hz), 1.49–1.59 (1H, m), 1.90–2.02 (2H, m), 2.13–2.32 (2H, m), 2.61–2.75 (2H, m), 2.88 (1H, t, *J* = 12.5 Hz), 3.91 (1H, d, *J* = 9.1 Hz), 6.81–7.00 (4H, m). ^13^C-NMR (CDCl_3_) δ (ppm) 20.9, 26.9, 27.0, 34.2, 35.8, 39.1, 41.2, 47.8, 54.9, 55.6, 108.3, 109.6, 115.2, 129.8, 129.9, 150.6, 160.6. Anal. Calcd for C_18_H_24_N_2_OS (316.46): C, 68.32; H, 7.64; N, 8.85; S,10.13%; Found: C, 68.45; H, 7.93; N, 8.50; S,9.89%.

*(1R,2R,7S,9R)-(10,10-Dimethyl-5-thia-3-azatricyclo[7.1.1.0^2,7^]undec-4-ylidene)-4-fluorophenylamine* (**26d**): *Method A*: 0.221 g (58%), *method B*: 0.232 g (61%); mp 201–205 °C, 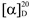 = +272.0 (*c* 0.25, MeOH), ^1^H-NMR (CDCl_3_) δ (ppm) 0.95 (3H, s), 1.25 (3H, s), 1.30 (1H, d, *J* = 10.6 Hz), 1.49–1.59 (1H, m), 1.90–2.02 (2H, m), 2.13–2.32 (2H, m), 2.61–2.75 (2H, m), 2.88 (1H, t, *J* = 12.5 Hz), 3.91 (1H, d, *J* = 9.1 Hz), 6.81–7.00 (4H, m). ^13^C-NMR (CDCl_3_) δ (ppm) 20.9, 26.9, 27.0, 34.2, 35.8, 39.1, 41.2, 47.8, 54.9, 55.6, 108.3, 109.6, 115.2, 129.9, 150.6, 160.6. Anal. Calcd for C_17_H_21_FN_2_S (304.43): C, 67.07; H, 6.95; N, 9.20; S, 10.53%; Found: C, 67.41; H, 7.13; N, 9.01; S 10.27%.

*(1R,2R,7R,9R)-(10,10-Dimethyl-5-thia-3-azatricyclo[7.1.1.0^2,7^]undec-4-ylidene)phenylamine* (**27a**): *Method A*: 0.186 g (52%), *method B*: 0.197 g (55%); mp 192–195 °C, 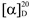 = +165.0 (*c* 0.25, MeOH), ^1^H-NMR (CDCl_3_) δ (ppm) 0.82 (3H, s), 1.25 (3H, s), 1.43–1.53 (1H, m), 1.67 (1H, d, *J* = 10.6 Hz), 1.91–2.16 (5H, m), 2.91–3.03 (2H, m), 3.44 (1H, d, *J* = 8.1 Hz), 6.95–7.28 (5H, m). ^13^C-NMR (CDCl_3_) δ (ppm) 20.1, 23.8, 27.3, 29.9, 33.8, 41.1, 41.5, 46.6, 58.1, 121.5, 123.0, 129.2, 145.2, 160.8. Anal. Calcd for C_17_H_22_N_2_S (286.43): C, 71.28; H, 7.74; N, 9.78; S, 11.19%; Found: C, 71.35; H, 7.83; N, 9.65; S, 11.17%.

*(1R,2R,7R,9R)-10,10-Dimethyl-5-oxa-3-azatricyclo[7.1.1.0^2,7^]undecane-4-thione* (**36**): *Method A*: 0.053 g (20%), *method B*: 0.055 g (21%); mp 176–179 °C, 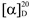 = +331.0 (*c* 0.25, MeOH), ^1^H-NMR (CDCl_3_) δ (ppm) 0.83 (3H, s), 1.33 (3H, s), 1.54 (1H, t, *J* = 12.5 Hz), 1.78 (1H, d, *J* = 10.7 Hz), 1.87–1.95 (1H, m), 2.03–2.12 (1H, m), 2.23–2.31 (1H, m), 2.40–2.53 (1H, m), 3.62 (1H, d, *J* = 10.2 Hz), 4.31 (1H, dd, *J* = 10.1, 12.5 Hz), 4.52 (1H, dd, *J* = 4.7, 10.0 Hz), 8.29 (1H, br s). ^13^C-NMR (DMSO–*d*_6_) δ (ppm) 19.6, 23.2, 23.5, 27.5,32.6, 41.6, 42.4, 43.1, 54.9, 75.6, 188.2. Anal. Calcd for C_11_H_17_NOS (211.32): C, 62.52; H, 8.11; N, 6.63; S, 15.17%; Found: C, 62.73; H, 8.46; N, 6.52; S, 15.01%.

*(1R,2R,7R,9R)-(3-Benzyl-10,10-dimethyl-5-thia-3-azatricyclo-[7.1.1.0^2,7^]undec-4-ylidene)phenylamine* (**28a**): *Method A*: 0.221 g (47%), *method B*: 0.231 g (49%); mp 108–109 °C, 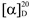 = +567.0 (*c* 0.25, MeOH), ^1^H-NMR (CDCl_3_) δ (ppm) 0.77 (3H, s), 1.20 (3H, s), 1.47 (1H, dt, *J* = 5.1, 13.6 Hz), 1.52 (1H, d, *J* = 10.8 Hz), 1.91–1.97 (1H, m), 2.04–2.14 (2H, m), 2.25–2.42 (2H, m), 2.88–2.99 (2H, m), 3.81 (1H, d, *J* = 8.1 Hz), 4.51 (1H, d, *J* = 16.4 Hz), 5.31 (1H, d, *J* = 16.6 Hz), 6.78–6.83 (2H, m), 6.94–6.99 (1H, m), 7.15–7.32 (7H, m). ^13^C-NMR (CDCl_3_) δ (ppm) 20.1, 24.2, 27.5, 31.1, 34.0, 35.8, 40.3, 40.4, 42.5, 49.0, 60.2, 122.7, 122.9, 126.7, 12.8, 128.7, 129.1, 140.2, 150.5, 152.6. Anal. Calcd for C_24_H_28_N_2_S (376.56): C, 76.55; H, 7.49; N, 7.44; S, 8.52%; Found: C, 76.86; H, 7.63; N, 7.21; S, 8.30%.

*(1R,2R,7R,9R)-3-Benzyl-10,10-dimethyl-5-oxa-3-azatricyclo[7.1.1.0^2,7^]undecane-4-thione* (**37**): *Method A*: 0.053 g (14%), *method B*: 0.060 g (16%); mp 94–97 °C, 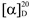 = +132.0 (*c* 0.25, MeOH), ^1^H-NMR (CDCl_3_) δ (ppm) 0.71 (3H, s), 1.23 (3H, s), 1.42–1.57 (2H, m), 1.83–1.91 (1H, m), 1.95–2.06 (2H, m), 2.20 (1H, t, *J* = 5.14), 2.43–2.51 (1H, m), 3.64 (1H, d, *J* = 9.6 Hz), 4.17 (1H, dd, *J* = 9.6, 12.2 Hz), 4.36 (1H, dd, *J* = 4.9, 9.6 Hz), 4.49 (1H, d, *J* = 16.2 Hz), 4.68 (1H, d, *J* = 16.2 Hz), 7.19–7.32 (5H, m). ^13^C-NMR (CDCl_3_) δ (ppm) 19.5, 23.5, 24.0, 27.8, 34.1, 40.6, 41.5, 41.6, 48.1, 57.1, 72.2, 127.3, 127.4, 128.9, 138.7, 155.8. Anal. Calcd for C_18_H_23_NOS (301.45): C, 71.72; H, 7.69; N, 4.65; s, 10.64%; Found: C, 71.98; H, 7.83; N, 4.39; S, 10.39%.

*(1R,2R,7S,9R)-Phenyl-(2,10,10-trimethyl-5-thia-3-azatricyclo[7.1.1.0^2,7^]undec-4-ylidene)amine* (**24a**): *Method A*: 0.240 g (64%), *method B*: 0.252 g (67%); mp 182–186 °C, 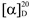 = –123.0 (*c* 0.25, MeOH), ^1^H-NMR (CDCl_3_) δ (ppm) 1.05 (3H, s), 1.28 (3H, s), 1.38 (1H, d, *J* = 12.5 Hz), 1.41 (3H,s), 1.69–1.98 (2H, m), 2.10–2.23 (2H, m), 2.42–2.53 (1H, m), 2.63 (1H, dd, *J* = 8.0, 12.8 Hz), 2.97 (1H, dd, *J* = 3.9, 12.9 Hz), 5.04 (1H, br s), 6.87–7.06 (3H, m), 7.22–7.30 (2H, m). ^13^C-NMR (CDCl_3_) δ (ppm) 24.1, 27.8, 28.2, 31.7, 33.4, 33.9, 40.3, 40.6, 55.3, 60.9, 122.7, 123.4, 129.2, 148.6, 158.4. Anal. Calcd for C_18_H_24_N_2_S (300.46): C, 71.95; H, 8.05; N, 9.32; S, 10.67%; Found: C, 72.19; H, 8.33; N, 9.11; S, 10.60%.

*(1R,2R,7S,9R)-2,10,10-Trimethyl-5-oxa-3-azatricyclo[7.1.1.0^2,7^]undecane-4-thione* (**34**): *Method A*: 0.028 g (10%), *method B*: 0.023 g (8%); mp 120–123 °C, 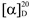 = +36.0 (*c* 0.25, MeOH), ^1^H-NMR (CDCl_3_) δ (ppm) 0.97 (1H, d, *J* = 11.1 Hz), 1.06 (3H, s), 1.31 (3H, s), ), 1.35 (3H, s), 1.79–1.87 (1H, m), 1.95–2.03 (2H, m), 2.14–2.42 (3H, m), 4.08 (1H, d, *J* = 11.0 Hz), 4.18 (1H, d, *J* = 10.7 Hz), 7.43 (1H, br s). ^13^C-NMR (CDCl_3_) δ (ppm) 20.9, 26.7, 27.2, 33.1, 34.1, 35.5, 39.3, 40.2, 41.2, 47.4, 55.1, 158.2. Anal. Calcd for C_12_H_19_NOS (225.35): C, 63.96; H, 8.50; N, 6.22; S, 14.23%; Found: C, 64.25; H, 8.79; N, 6.01; S, 13.88%.

*(1aR,2aR,6aS,7aS)-Phenyl-(1,1,6a-trimethyloctahydro-4-thia-6-azacyclopropa[b]naphthalen-5-ylidene)amine* (**25a**): *Method A*: 0.225 g (60%), *method B*: 0.233 g (62%); mp 157–160 °C, 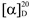 = −18.0 (*c* 0.25, MeOH), ^1^H-NMR (CDCl_3_) δ (ppm) 0.71–0.83 (2H, m), 0.98 (3H, s), 1.07 (3H, s), 1.22 (1H, dd, *J* = 4.5, 15.8 Hz), 1.28 (3H, s), 1.53–1.71 (2H, m), 1.89–2.01 (2H, m), 2.65 (1H, d, *J* = 3.4, 12.4 Hz), 3.29 (1H, d, *J* = 3.5, 11.7 Hz), 7.02–7.12 (3H, m), 7.28–7.34 (2H, m). ^13^C-NMR (CDCl_3_) δ (ppm) 15.6, 17.6, 18.2, 19.6, 21.8, 28.8, 28.9, 31.6, 35.4, 52.1, 123.2, 123.4, 123.9, 129.3, 155.0. Anal. Calcd for C_18_H_24_N_2_OS (300.46): C, 71.95; H, 8.05; N, 9.32; S,10.67%; Found: C, 72.20; H, 8.19; N, 9.02; S, 10.54%.

#### 3.2.4. Alternative Procedure for the Synthesis of 2-Thioxo-1,3-oxazines **35** and **36**

The respective amino alcohol **16** or **17** (1.18 mmol) in dry toluene solution (20 mL), TEA (3.54 mmol) and thiophosgene (0.181 mL, 2.36 mmol) was stirred at room temperature for 6 h. Next, the solvent was evaporated off under reduced pressure and the residue was purified by flash column chromatography on silica gel; elution with *n*-hexane/EtOAc (4:1) gave 2-thioxo-1,3-oxazine **35** or **36**.

#### 3.2.5. General Procedure for the Synthesis of 2-Imino-1,3-thiazines **42** and **43**

*Method A*: The respective thiourea (1.25 mmol) **38** or **39** in THF solution (12 mL) and CDI (0.306 g, 1.88 mmol) was heated to reflux for 3 h. Next, the solvent was evaporated off under reduced pressure and the residue was purified by flash column chromatography on silica gel; elution with *n*-hexane/EtOAc (4:1) gave the pure 2-imino-1,3-thiazines **42** or **43**.

*Method B*: A CEM Discover 10 mL vial containing the respective intermediates (0.63 mmol) **40** or **41** in THF solution (12 mL) was heated to reflux for 3 h. Next, the solvent was evaporated off under reduced pressure and the residue was purified by flash column chromatography on silica gel; elution with *n*-hexane/EtOAc (4:1) gave the pure 2-imino-1,3-thiazines **42** or **43**.

*(Hexahydrocyclopenta[d][1,3]thiazin-2-ylidene)phenylamine* (**42**): 0.218 g (75%); mp 180–181 °C (Lit. [33] 180–181 °C), ^1^H-NMR (CDCl_3_) δ (ppm): 1.47–1.71 (3H, m), 1.74–2.10 (3H, m), 2.52–2.66 (1H, m), 2.77 (1H, dd, *J* = 9.3, 12.5 Hz), 2.92 (1H, dd, *J* = 4.5, 12.5 Hz), 3.81–3.93 (1H, m), 6.95–7.10 (3H, m), 7.26–7.34 (2H, m). ^13^C-NMR (CDCl_3_) δ (ppm): 24.0, 31.4, 31.7, 35.7, 39.7, 57.3, 122.5, 123.4, 129.2, 148.5, 157.6. Anal. Calcd for C_13_H_16_N_2_S (232.34): C, 67.20; H, 6.94; N, 12.06; S, 13.80%; Found: C, 67.47; H, 6.81; N, 12.30; S, 13.42%.

*(Octahydrobenzo[d][1,3]thiazin-2-ylidene)phenylamine* (**43**): 0.222 g (72%); mp 183–186 °C (Lit.[33] 187–188 °C), ^1^H-NMR (CDCl_3_) δ (ppm): 1.30–1.81 (7H, m), 1.83–1.97 (1H, m), 2.05–2.15 (1H, m), 2.81 (1H, dd, *J* = 4.8, 12.4 Hz), 3.12 (1H, dd, *J* = 4.1, 12.5 Hz), 3.57–3.65 (1H, m), 6.98–7.10 (3H, m), 7.21–7.30 (2H, m). ^13^C-NMR (CDCl_3_) δ (ppm): 21.0, 24.7, 26.4, 32.2, 32.6, 34.3, 52.5, 122.8, 123.5, 129.3, 146.1, 156.4. Anal. Calcd for C_14_H_18_N_2_S (246.34): C, 68.25; H, 7.36; N, 11.37%; Found: C, 68.31; H, 7.51; N, 11.02%.

#### 3.2.6. Determination of Antiproliferative Activities

Antiproliferative effects against four human cancer cell lines were determined as published recently [36]. Briefly, HeLa (cervix adenocarcinoma), MCF7 (breast adenocarcinoma), A2780 (ovarian carcinoma) and A431 (skin epidermoid carcinoma; all cell lines purchased from ECACC; Salisbury, UK) cells were cultivated in minimal essential medium (Sigma-Aldrich, Budapest, Hungary) supplemented with 10% foetal bovine serum, 1% non-essential amino acids and an antibiotic-antimycotic mixture.

Near-confluent cells were seeded into a 96-well plate (5000 cells/well) and, after overnight standing, the medium (200 µL) containing the tested compound (at 10 or 30 µM) was added. Following a 72-h incubation in a humidified atmosphere of 5% CO_2_ at 37 °C, the living cells were assayed by the addition of 20 µL of 5 mg/mL MTT [3-(4,5-dimethylthiazol-2-yl)-2,5-diphenyltetrazolium bromide] solution [37]. During a 4-h contact period, the MTT was converted by intact mitochondrial reductase and precipitated as blue crystals. The medium was then removed, the precipitated formazan crystals were solubilized in DMSO (100 µL) during a 60-min period of shaking at 25 °C, and the absorbance was read at 545 nm with a microplate reader. Wells with untreated cells were utilized as controls. All *in vitro* experiments were carried out on two microplates with at least five parallel wells. Stock solutions of the tested substances (10 mM) were prepared with DMSO. The DMSO concentration (0.3%) of the medium did not have any significant effect on cell proliferation. Cisplatin was used as reference compound.

#### 3.2.7. X-ray Crystallographic Studies

Crystallographic data were collected at 123 K for **31c** and **31d** by using a Bruker Nonius-Kappa CCD diffractometer with an APEXII area detector and graphite-monochromatized Mo-K_α_ radiation (λ = 0.71073 Å), as reported earlier [38].

Crystal data for **31c**: C_21_H_25_FN_4_O_2_S, *M_r_* = 416.51, triclinic, space group *P-1* (no. 2), *a* = 8.5831(5), *b =* 10.7338(6), *c* = 11.9244(5) Å, *α* = 86.414(3)°, *β* = 69.835(3)°, γ = 85.729(3)°, *V* = 1027.59(9) Å^3^, *T* = 123 K, *Z* = 2, μ(Mo-*K*_α_) = 0.192 mm^−1^, 6132 unique reflections (*R_int_* = 0.0211) which were used in calculations. The final *R1* (for the data with *F^2^* > 2δ(*F^2^*) was 0.0426 and *wR2*(*F^2^*) (all data) was 0.1034.

Crystal data for **31d**: C_22_H_28_N_4_O_3_S, *M_r_* = 428.54, monoclinic, space group *C2/c* (no. 15), *a* = 23.1162(6), *b =* 8.7978(3), *c* = 21.4232(5) Å, *β* =92.4232(5)°, *V* = 4352.8(2) Å^3^, *T* = 123 K, *Z* = 8, μ(Mo-*K*_α_) 0.180 mm^−1^, 7979 unique reflections (*R_int_* = 0.0279) which were used in calculations. The final *R1* (for the data with *F^2^* > 2δ(*F^2^*) was 0.0454 and *wR2*(*F^2^*) (all data) was 0.1063.

The structures were solved by direct methods by use of the SHELXS-97 program [39], and full-matrix, least-squares refinements on *F^2^* were performed by use of the SHELXL-97 program [39]. The CH hydrogen atoms were included at fixed distances from their host atoms with the fixed displacement parameters. The NH hydrogen atom positions were refined with the fixed displacement parameters. In **31d**, the methoxy C atom has two different orientations in a 1:1 ratio (due to packing reasons). The graphics were drawn with ORTEP3 for Windows [40]. The depositions numbered CCDC 1000179 (for **31c**) and CCDC 1000178 (for **31d**) contain the supplementary crystallographic data for this paper. These data can be obtained free of charge at www.ccdc.cam.ac.uk/conts/retrieving.html (or from The Cambridge Crystallographic Data Centre via www.ccdc.cam.ac.uk/data_request/cif).

## 4. Conclusions

In conclusion, we have developed a mild and efficient method for the synthesis of 2-imino-1,3-thiazines by the ring closure of thiourea adducts of 1,3-amino alcohols in the presence of CDI under microwave condition. Besides the main product 1,3-thiazines, the formation of 2-thioxo-1,3-oxazines as side-products was observed. The ring-closure process was extended to cycloalkane-based γ-hydroxythioureas and the method developed for the synthesis of 2-imino-1,3-thiazines containing an acid-sensitive structure proved to be comparable to those reported in the literature. The resulting 1,3-thiazines exert marked antiproliferative action on a panel of human cancer cell lines.
